# Allocating resources to support universal health coverage: development of a geographical funding formula in Malawi

**DOI:** 10.1136/bmjgh-2020-002763

**Published:** 2020-09-15

**Authors:** Finn McGuire, Paul Revill, Pakwanja Twea, Sakshi Mohan, Gerald Manthalu, Peter C. Smith

**Affiliations:** 1 Centre for Health Economics, University of York, York, UK; 2 Department of Economics, University of York, York, United Kingdom; 3 Department of Planning and Policy Development, Ministry of Health, Lilongwe, Malawi; 4 Imperial College Business School, Imperial College, London, United Kingdom

**Keywords:** health economics, health policy, health systems

## Abstract

**Background:**

Universal health coverage (UHC) requires that local health sector institutions—such as local authorities—are properly funded to fulfil their service delivery commitments. In this study, we examine how formula funding can align sub-national resource allocations with national priorities. This is illustrated by outlining alternative options for using mathematical formula to guide the allocation of national drug and service delivery budgets to district councils in Malawi in 2018/2019.

**Methods:**

We use demographic, epidemiological and health sector budget data with information on implementation constraints to construct three variant allocation formulae. The first gives an equal per capita allocation to each district, and is included as a baseline to compare alternatives. The second allocates funds to districts using estimates of the resources required to provide Malawi’s essential health package of priority cost-effective interventions to the full population in need of each intervention. The third adjusts these estimates to reflect a practicable level of attainable coverage for each intervention, based on the current configurations of health services and demand for interventions.

**Findings:**

Compared with current district allocations, not underpinned by an explicit formula, the formulae presented in this study suggest sizeable shifts in the allocations received by many districts. In some cases, the magnitude of these shifts exceed 50% reductions or doubling of district budgets. The large shifts illustrate inequities in the current system of budget allocation and the potential improvements possible.

**Conclusion:**

The use of mathematical formulae can guide the efficient and equitable allocation of healthcare funds to local health authorities. The formulae developed were facilitated by the existence of an explicit package of priority interventions. The approach can be replicated in wide range of countries seeking to achieve UHC.

**Key questions**

**What is already known?**

- Prioritisation of limited healthcare resources is even more important in the constrained settings of low- and middle-income countries (LMICs).
- Many LMICs have outlined health benefits packages (HBPs) to guide healthcare resources towards priority set of healthcare interventions.
- However, resource allocation formulae are rarely aligned with these prioritised healthcare interventions.

**What are the new findings?**

- This study examines the main considerations which should be accounted for when aligning resource allocation formulae with HBPs and uses a case study of Malawi to illustrate how this can be straightforwardly done.
- Using such methods ensures countries are guided by their national priorities but also account for substantive equity considerations at subnational level.

**What do the new findings imply?**

- LMICs which have explicitly outlined HBPs can use these—as well as the data used for their development—as the basis for health sector resource allocations.
- Resource allocation formulae linked to HBPs can assist in equitably delivering nationally defined priority healthcare services. Such formulae can increase transparency in decision-making to ensure geographical regions receive a fair distribution of health resources with which to provide health services to the local population.

## Introduction

A fundamental requirement of universal health coverage (UHC) is that local health sector institutions—such as local governments and health authorities—are given the funding necessary to fulfil their service delivery commitments. This paper focusses on one approach to funding UHC, using mathematical formulae to determine the magnitude of funds directed towards local health agencies, with reference to district councils in Malawi. The use of such formulae has become widespread in health systems across the world and offers enormous scope for ensuring that funding is aligned with UHC objectives.^1–3^

There are three broad reasons for adopting a formulaic approach towards creating local budgets, reflecting efficiency, equity and political objectives.^4^ The efficiency argument seeks to ensure that the available national funds are distributed in line with national policy objectives embodied in the chosen approach to UHC. Many low- and middle-income countries (LMICs) suffer large intra-country inequities in access to health services, hindering progress towards achieving UHC.^5–7^ Systems of formula funding intrinsically seek to promote equity, in the sense that all citizens in medical need of a specified service should be able to secure equal opportunity of access to that service.^4^ Finally, the formulaic approach to funding can serve certain political objectives attached to the distribution of funds, such as allowing the criteria for funding to be set out explicitly; promoting accountability; avoiding the need for case by case scrutiny of budgets; and to provide a non-partisan solution to political conflicts.^4 8 9^ The relevance of resource allocation formulae in accelerating progress towards UHC is manifest.

In high-income countries, formula funding has become the dominant mechanism for devolving health system finances. Typically, a ‘capitation’ approach is adopted, based on measures of the size and characteristics of a locality’s population, adjusted for risk factors such as levels of disease and poverty; although the level of sophistication used varies markedly between countries.^10 11^ Increasingly the approach is also being adopted in LMICs, however, in practise many countries continue to rely on historical allocations.^12^ Health system goals of efficiency and equity are similar in many countries, including LMICs, and require allocations based on need rather than historical supply.^13^

Some LMICs have attempted to move beyond historical allocations, towards the distribution of resources based on indicators that attempt to capture variation in ‘need’. In a review, Anselmi *et al* (2015) identify eight resource allocation studies (six in Africa) and highlight use of population weighting by demographic, socioeconomic and health status characteristics including mortality as common components of allocation formulae.^14^ All of the African cases identified were produced by EQUINET—the Regional Network on Equity in Health.^12^ These formulae can be simplistic, such as those based solely on demographic-adjusted population, or complex.

Uganda, for instance, introduced a formula to allocate its health budget to districts based on an index of demographic adjusted population, a human development index (including per capita income, life expectancy and school enrolment ratios) and local donor spending.^15^

Despite this, three main barriers have been identified which continue to slow progress towards UHC; availability of resources, over-reliance on out-of-pocket payments and inefficient and inequitable use of available resources.^16^ While resource allocation formulae intend to address the last barrier, they are not the only mechanism in doing so. Another approach an increasing number of LMICs are adopting is to design explicit health benefits packages (HBPs). HBPs are, broadly, a defined list of services that will be publicly funded and are increasingly recognised as an important component of priority-setting processes in LMICs.^17^ Some studies have highlighted that, rather than formulae being based on proxies of need, they could instead be more explicitly linked to HBPs.^18 19^ We contend that such a linking of resource allocation formulae to predefined HBPs is highly advantageous in operationalising national objectives and in matching resources to the relative need for healthcare services within LMICs. We show how the basic principles of resource allocation formulae development can be adapted to realise such a linkage.

Therefore, the primary objective of the paper is to demonstrate how linking subnational resource allocation to pre-existing national health sector policies, specifically clearly defined HBPs, may be practically implemented. In doing this, we show how this method of resource allocation formula development requires a number of normative choices. We illustrate the advantages and disadvantages of methodological decisions stemming from these choices. We present Malawi as a case study to illustrate how such methods can be applied and implemented.

This method of resource allocation formula development impacts a number of general issues that remain important for resource allocation formula. A number of authors have noted the variations in alternative sources of funding, in particular from donors and non-governmental organisations, complicate governments’ funding decisions.^15 20 21^ Although vertical donor financing arrangements can be an obstacle to formula funding, donors also have the potential to play an important role if they can support the development and use of formulae.^12^ Other obstacles include data limitations and varying capacity of districts to absorb funding, leading some authors to conclude formula funding should be used in combination with other initiatives to strengthen national and local purchasing and planning, including finding ways to strengthen local health systems.^12 22^ Finally, it has been recognised that cross-boundary flows of populations seeking care may also need to be considered for formula development.^23^

### Malawi’s health sector and budget

Malawi, like many LMICs, has pursued a policy of decentralisation in its delivery of health services. The Ministry of Local Government and Rural Development (MoLGRD) is responsible for the delivery of health services to Malawi’s 17.56 million population at the local level through 28 district councils.^24^ In 2018/2019 the total health budget comprised the third largest share of the total government budget; amounting to ~US$183 million (US dollar) (MWK128 billion (Malawi Kwacha)),^25^ of which ~US$77 million (MWK56 billion) (44%) is allocated to districts.^26^

There are four main budgets used to finance healthcare; for drugs, other recurrent transactions (ORT), personal emoluments (PE) and development (eg, capital expansion). At 64%, PE constitutes a majority of district’s health sector budget, followed by drugs and ORT, at 23% and 13%, respectively.^26^ The MoLGRD is responsible for the allocation of the drug and ORT budgets to districts, in consultation with the Ministry of Health (MoH). PE and development budgets are decided by other central Government departments. District councils then allocate their budgets between the district hospital, health centres, dispensaries, village clinics, community health posts and outreach services. Tertiary care (central hospitals) are not devolved and are funded separately. In addition to the national budget, health financing at district level benefits from donor finances—particularly for interventions targeted at nutrition, HIV/AIDS, malaria and tuberculosis—and some local level revenue collection.

**Figure 1:**
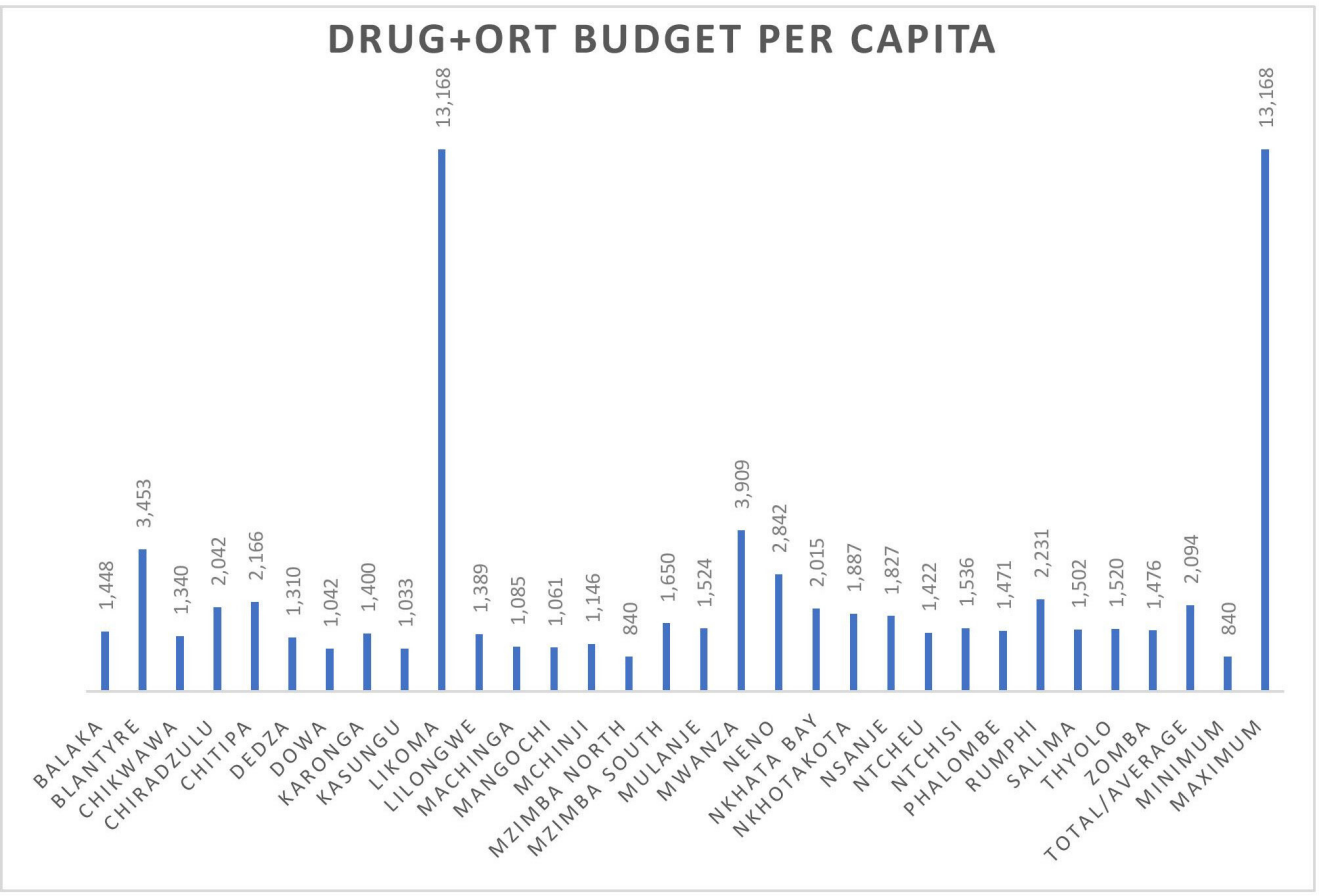
Per capita district allocation (2018/2019). ORT, other recurrent transactions.

Figure 1 shows the allocation of the drug and ORT budget across districts in per capita terms for the 2018/2019 fiscal year. Currently, historic allocations are used with relative allocations to each council rarely change materially. Per capita allocations vary extremely widely across districts; Likoma Island receives ~US$17.94 (MWK13 168) per capita while Mzimba north receives ~US$1.14 (MWK840) per capita. The average district allocation per capita is ~US$2.85 (MWK2094). Although this does not necessarily indicate that the distribution fails the principle of a needs-based allocation, as the healthcare needs of individuals are not homogeneous. Due to the years of reliance on historical allocations it is unclear how well the current allocation mechanism reflects the relative needs of districts within Malawi. Such an allocation mechanism, based on historical allocations, results in perpetuating existing inequities.

In 2017 a health benefits package—known as the essential health package (EHP)—was revised to support service implementation towards UHC.^27 28^ The national-level EHP is intended to concentrate the scarce health sector resources of Malawi on a number of key health interventions for which access is free at the point of delivery. The revised EHP includes 97 interventions, selected according to a number of criteria, including estimated cost-effectiveness (online supplementary appendix 1). Despite a national prioritisation process taking place in the revision of the EHP, no mechanism currently exists to ensure subnational budgetary allocations are made on the basis of districts’ relative EHP-related need.

10.1136/bmjgh-2020-002763.supp1 Supplementary data

## Methods

This study develops mathematical formulae to inform the distribution of the ORT and drug budgets, totalling ~US$31 million in 2018/2019, to districts in Malawi for the delivery of services contained in the EHP. The formula seeks to reflect local variations in the need for those services. Note that the formula addresses only the distribution of resources to districts. How districts use these resources to purchase services from providers (health centres, district hospital, and so on) is subject to local discretion, and lies outside the scope of this work. Our study focusses on these budgets due to local institutional arrangements which imply that the PE and development budgets fall outside of the remit and influence of the MoH, are not subject to the same degree of decentralisation, and, therefore, are unsuitable for inclusion in any practical resource allocation formulae. Further, only government funds are included for allocation by the formulae, thereby minimising the potential for external constraints preventing realisation of implied allocations. Such considerations are important in ensuring any formulae developed are realised and are idiosyncratic to each case (Specifically, they are not part of the Intergovernmental Fiscal Transfer Formula. See accompanying paper by Twea *et al* (2020)^2^,^9^ for further information). The implications of these issues are outlined in the discussion.

We present three variant resource allocation formulae. Each variant is intended to capture progressively more details on factors contributing to a district’s expected annual expenditure on EHP provision. The first, is based on unadjusted population size and represents a population baseline against which to compare the allocations implied by the latter two formulae. The second variant adjusts resource allocations for total EHP intervention ‘need’ seeking to capture district spending need as accurately as possible, regardless of access barriers that may constrain a district’s ability to use the funds allocated as intended. The third variant augments the EHP intervention need allocations by accounting for service delivery constraints. It incorporates realistic limits to actual utilisation for each service, based on estimates of Malawi’s recent implementation performance across interventions.

### Crude population allocation

At its most basic, capitation resource allocation formulae account only for the size of the population for which the local agency is responsible. This scenario was used as a benchmark against which resource reallocations based on relative EHP ‘need’ were assessed. District-level populations were calculated using the 2018 district population projections of the Population and Housing Census (2008) (It should be noted that a 2018 census was in the final stages of development during the preparation of this manuscript. Once available it should be used to update district population and subsequent formula allocation estimates.). The 2018/2019 total drug and ORT budgets were divided by the aggregate population of Malawi to give the per capita budget allocation. These figures were then multiplied by each district-level population resulting in districts receiving a budget proportional to its share of the aggregate national population. This methodology, in effect, attaches a uniform need for expenditure on EHP services to each citizen, regardless of other characteristics. Given the available budget, a value of MWK1265 is allocated to each citizen. In practice, healthcare needs of individuals vary considerably depending, for instance, on age, morbidity or social factors. This formula is, therefore, not reflective of real variations in expenditure need across districts, but is instead used as a benchmark against which to measure the redistributive consequences of the other variants.

### EHP intervention need (full coverage)

The second variant takes as its departure point the pre-established interventions which Malawi has agreed should be provided free at the point of access to all citizens. The key principle of the formula is to attempt to reflect local variations in spending need specifically on those services. This requires predicting district level demand associated with the provision of EHP services. The allocation formula, in that sense, reflects not just the relative health needs of districts, but also the normatively determined priorities of the country from previously outlined government policy. Without reference to the EHP any formulae risks undermining national priorities and inhibit the implementation of health benefits packages.

A bottom-up approach was taken to calculating the EHP-related healthcare need and associated expected costs in order to align the allocation of resources to these. This involved disaggregating the EHP by the number of interventions within the package. For each intervention an associated unit cost of delivery per patient was calculated. Finally, for each district, an estimate of the number of people expected to need the intervention is calculated. Each district’s expected annual expenditure is then the aggregation of the district annual expenditure on each EHP intervention. Once this expected annual EHP-related expenditure is calculated for each district, national budget data can be used to calculate the relative resource allocations across districts.

Each district’s expected annual expenditure on each EHP intervention is, therefore, a factor of two calculated variables; (1) the number of people expected to need the intervention and (2) the unit cost of providing the intervention.

Unit cost data for each of the 97 EHP interventions was available from the recently costed Health Sector Strategic Plan II (2017 to 2022). An inputs-based approach is used to cost each intervention calculated by multiplying the unit cost of drugs and commodities required for each intervention by the units required per person per year and the proportion of patients who receive each commodity. This provides an average drug and commodity unit cost per intervention. An important aspect of resource allocation formulae is they reflect only variations in expected expenditure related to the budgets which they intent to allocate. Given the allocation formula was designed to apply only to the drug and ORT budgets it was not deemed relevant to attempt to capture possible variations in other costs, for example, human resource, which may occur across districts.

The expected number of individuals within a district requiring treatment with an EHP intervention is calculated as the product of the relevant population target group (those ‘at risk’) and the conditional probability of requiring the intervention for individuals within that target population. Various data sources were used to calculate information on the expected number of cases per year in districts. Each EHP intervention has an explicitly outlined ‘target population’, indicating the relevant subpopulation whom could potentially require a given intervention. For instance, the target population for management of eclampsia within a district is the number of pregnant women within the district. Data on target populations figures across districts was taken from various sources of demographic and epidemiological data including the Demographic and Health Survey (2015/2016), Integrated Household Survey (2016/2017) and District Health Information System. Data on the proportion of the target population actually requiring the intervention per annum—the ‘population in need’—is based on the incidence and prevalence of conditions as well as country treatment guidelines (This was also provided from the Health Sector Strategic Plan (2017 to 2022).). Therefore, the population in need (PIN) is the proportion of the target population—in this case pregnant women—that is expected to suffer from and therefore require treatment for pre-eclampsia, that is, 2.18% of pregnant women nationally (A subsample of target population size calculations are contained in online supplementary appendix 2 and for intervention total estimates in an accompanying report 18. McGuire, F; Revill, P; Twea, P; Mohan, S; Manthalu, G; Smith, PC; *Recommendations for the development of a health sector resource allocation formula in.* Malawi 2018, Centre for Health Economics, University of York (CHE Research Paper No. 159): York, UK.). The expected number of people in a district requiring pre-eclampsia treatment per annum would, therefore, be this proportion of the estimated number of pregnant women in the district.

Therefore the total district cost of providing an EHP intervention is the product of the target population and PIN multiplied by the unit cost of providing the intervention. All inputs and calculations are shown in online supplementary appendix tables S1 and S2.

### Equation 1: methodology for calculating expected intervention expenditure (full coverage)

Numbers patients requiring intervention X Cost per person per year by intervention = Total expected expenditure on drugs and commodities for intervention

where

Numbers patients requiring intervention = Target population size (#) X Target population in need (PIN) of the intervention (%)

District’s total EHP-related spending need is calculated as the aggregation of the total expected expenditure on all EHP interventions. As mentioned, because the drug and ORT budgets are set and insufficient to fully cover EHP-related expenditure need nationally, each districts proportion of total spending need is calculated with allocations of the available budget made in accordance to this relative proportion.

$$TC_{i}=\sum_{j=1}^{97}IC_{ji}$$

$${PC}_{i}=\frac{{TC}_{i}}{\sum_{i=1}^{28}{TC}_{i}}x100$$

$$District\;i\;total\;EHP\;budget=PC_{i}\;x\;TB$$

Where ${IC}_{ji}$ is cost of intervention j in district i, ${TC}_{i}$ is total EHP cost in district i, $\sum_{i=1}^{28}{TC}_{i}$ is the total national EHP cost, ${PC}_{i}$ is the proportion of district i EHP cost and TB is the total national ORT and drug budget.

### EHP intervention need (realistic coverage)

The third scenario builds on the second by removing the assumption that all patients requiring an EHP intervention are treated. Instead, since some interventions are more challenging to deliver at scale than others, an estimate of the national average attainable coverage level for each intervention is applied to all districts, expressed as a proportion of the total estimated need for the intervention (as calculated in the full coverage scenario.). The attainable coverage level is the estimated proportion of individuals in need of the intervention who could receive it on the basis of current level of implementation coverage in the national healthcare system.^30^ Therefore, attainable coverage explicitly references the current levels of unmet need across EHP interventions. These measured rates of unmet need are used to adjust to fit district allocations within the aggregate budget, calculating the funding required if national levels of coverage for each intervention were to apply in each district. This removes the assumption that the provision of all services should be reduced by an equal proportion from the ideal level. We drew on national level estimates of actual coverage levels by intervention used in the development of the Malawian EHP.^27^ This replaces the assumption of full coverage with an assumption that attainable coverage for each intervention is constant across all districts, but varies by intervention type. Therefore, any variations in allocations compared with the full implementation scenario will originate from different epidemiological patterns of diseases across different districts, not from differences in district coverage levels for EHP interventions.

Utilisation constraints may operate both on the supply side, in terms of the health system’s capacity to deliver an intervention, and on the demand side, through the limited uptake by those with capacity to benefit from an intervention. We make no assumptions regarding the sources of the utilisation constraints, which may vary across districts. This approach aims to adjust allocations according to an estimate of ‘realistic’ coverage for each intervention at a national level, and retains the principle of an equitable allocation by assuming that those coverage levels are replicated in each district. This can be viewed as a mild method of capturing potential differences in district-level absorptive capacities, informed by both the budgets being allocated and contextual knowledge. Absorption of drug and ORT budgets is not an issue in Malawi; in fact, between 2012/2013 and 2017/2018, high levels of recurrent expenditure crowded out the space for development expenditure,^31^ making it all the more important to better project healthcare expenditure needs and improve budget compliance for resource use efficiency and equity.

### Equation 2: methodology for calculating expected intervention expenditure (realistic coverage)

Numbers patient requiring intervention X Cost per person per year by intervention = Total expected expenditure on drugs and commodities for intervention

where

Numbers patients requiring intervention = Target population size (#) X Target population in need (PIN) of the intervention (%) Realistic coverage of intervention (%)

For both the ‘full coverage’ and ‘realistic coverage’ scenarios, a district’s budget requirement to deliver the entire EHP is the sum of the expenditure needs required across all interventions. This leads to a national aggregate budget requirement greatly in excess of the national budget currently available. Consequently, without additional resource mobilisation, the EHP interventions will not be provided to all those who require them through government financing alone. This issue is an inevitable consequence of aspirational healthcare planning and severely limited resources, faced by most LMICs. We address this by scaling down each district’s expenditure needs assessment by a uniform proportion in order to adhere to the national budget available. This approach implicitly assumes that, given the national budget shortfall, each district receives an equal proportionate reduction in the allocation implied by the funding formula. Our approach is informed by equity considerations and the idea that no singular district should be penalised for national budgetary shortfalls. The formulae therefore reflects the relative healthcare expenditure needs of individuals and districts rather than the absolute expenditure requirement. The size of the scaling down is substantive, with the total cost of providing the HBP under ‘full coverage’ and ‘realistic coverage’ scenarios of ~US$179 million and ~US$122 million, respectively. Given the ~US$31 million available for allocation this implies the average district only being provided between 17% to 25% of the funds required.

### Patient and public involvement

Due to the nature of the work, regarding health sector budgets, involving patients was not feasible. Although it was deemed inappropriate to involve the public during the course of the research, the methods proposed in the study do not preclude patient involvement in the decision-process. Additionally, we encourage the methods and results of any resource allocation formulae to be disseminated widely and made publicly available.

## Results

**Figure 2:**
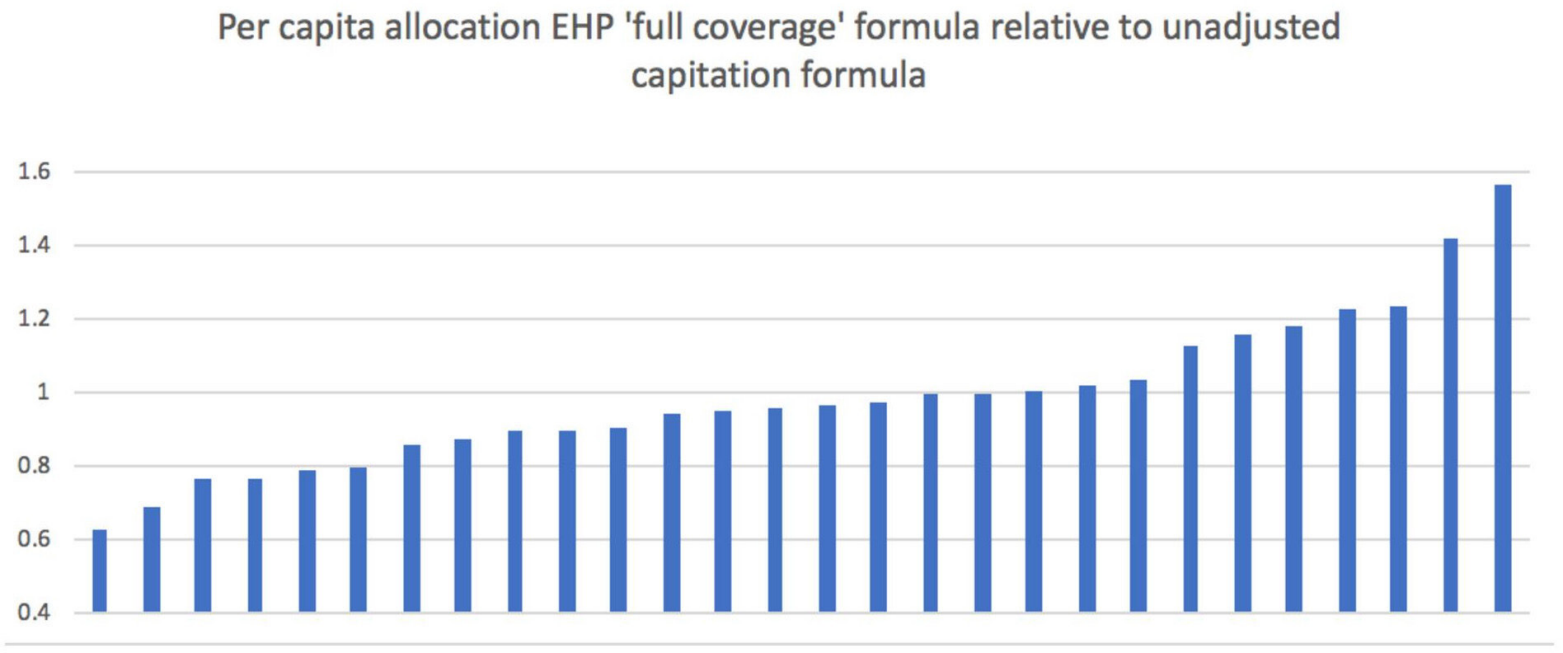
District per capita allocations with EHP ‘full coverage’ formula relative to per capita allocations with unadjusted capitation formula. EHP, essential health package.

**Figure 3:**
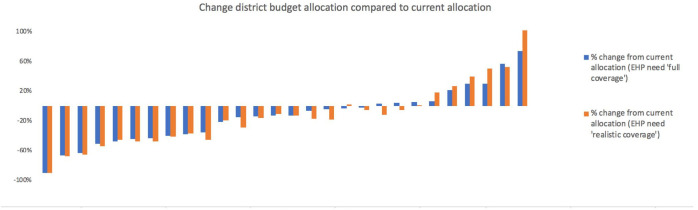
Shift in budgetary share implied by use of the EHP formula (% change). EHP, essential health package.

Figure 2 presents per capita allocations implied by adoption of the second formula variant (EHP need ‘full coverage’) relative to the uniform per capita allocations across districts from the unadjusted population allocation (equal to 1.0). It varies across districts from about two-thirds to about 1.5 times the national average. This makes explicit the cross district variation in the expected health expenditure per capita based on district variation in EHP-related disease burdens. While variation in total budget allocations may exist due to district population disparities, figure 2 makes clear that certain districts populations are in much greater need of EHP-related healthcare services, irrespective of population levels. This is perhaps unsurprising, as communicable diseases still contribute much of the disease burden faced by Malawi, which would be expected to lead to concentrated pockets of need more than non-communicable illnesses. Therefore, there are large inequalities across districts in terms of EHP-related disease burdens which this formula seeks to account for. Figure 3 shows the resulting percentage changes in moving from current district allocations to those implied by the EHP need formulae. There are very large reductions (over 50%) in four districts, and a near doubling of allocations to one district. Such very large swings could not be contemplated in a single fiscal year, but they do indicate the highest priority districts for future budgetary changes.

It is noteworthy that the current district allocations are providing comparatively high allocations to some districts with relatively low EHP-related disease burdens and populations. This could reflect other legitimate factors such as true variation in costs of healthcare delivery. Targeted donor funds or illegitimate influences on allocations such as differences in political power. Although, with the current data, it is not possible to confirm whether the differences between current allocations and the (close to) ‘true’ allocative need (reflected by the EHP formulae) are driven by legitimate or illegitimate factors, such a comparison does provide a simple sense check. More specifically, if both EHP need based allocation and unadjusted capitation formulae are greater than (or less than) current allocations, the difference in allocations would need to be justified by offsetting legitimate factors such as lower (higher) cost of healthcare delivery of equal magnitude.

**Figure 4:**
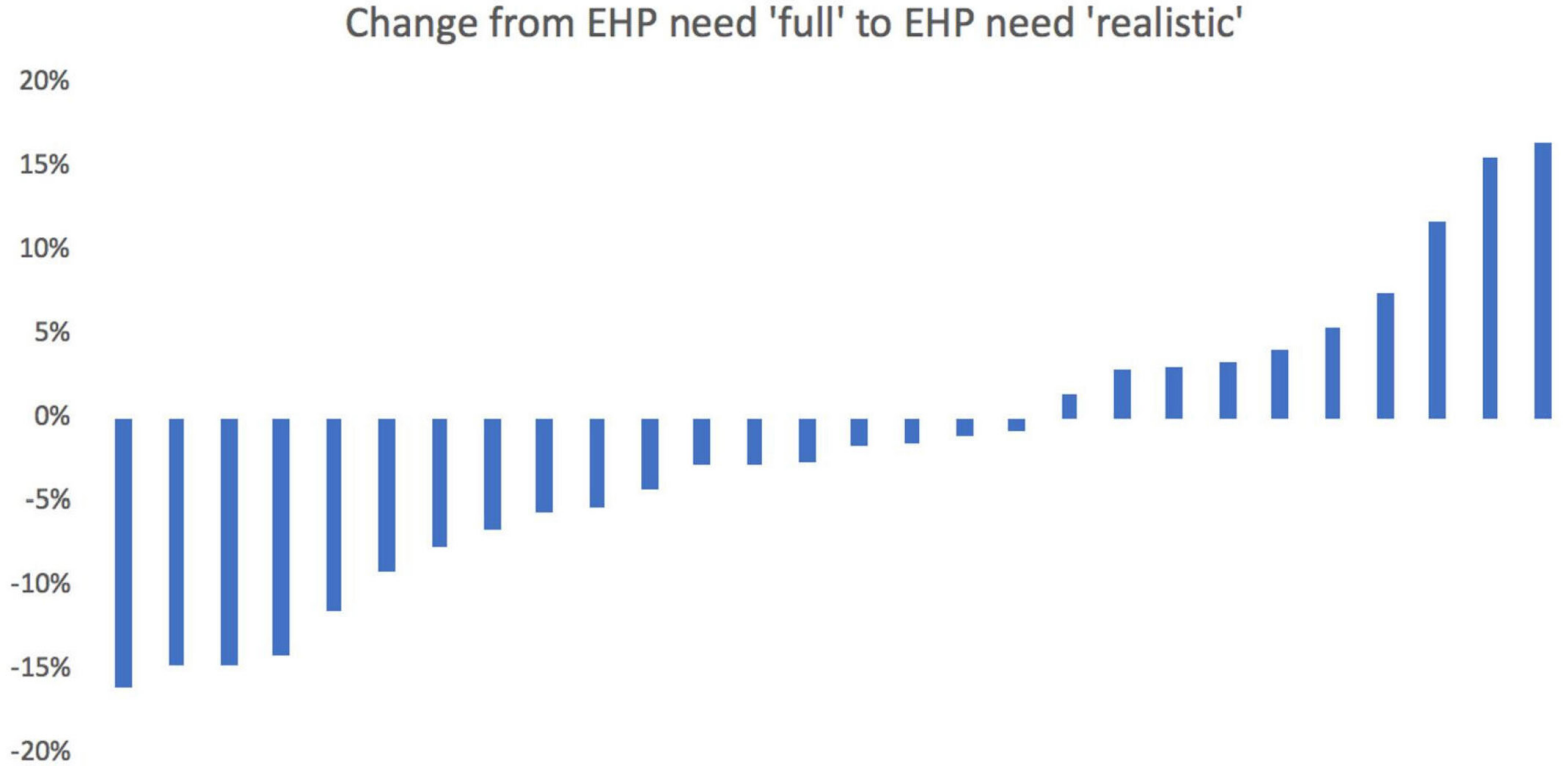
Shift in budgetary share between ‘full’ EHP need formula and ‘realistic’ EHP need formula (% change). EHP, essential health package.

The percentage changes to district allocations that occur when moving from the second variant to the third variant, with more ‘realistic’ assumptions about specific intervention coverage, are shown in figure 4. This formula variant implies more moderate reallocations with changes ranging from a decrease of 16% to an increase of 16.5%, compared with the unadjusted capitation variant.

**Figure 5:**
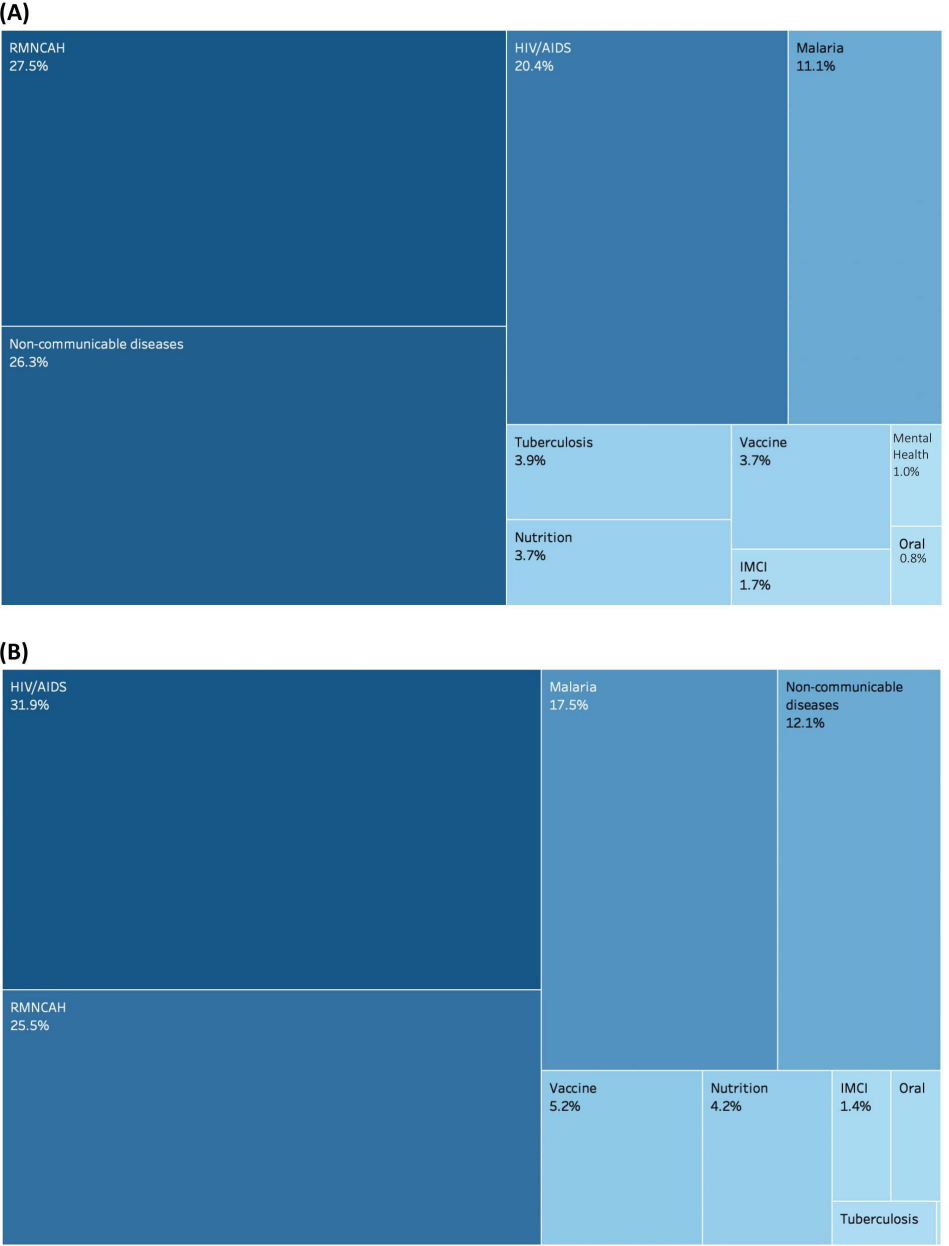
(A) Relative weight of national budget allocations to disease areas in the EHP ‘full coverage’ formula and (B) relative weight of national budget allocations to disease areas in the EHP ‘realistic coverage’ formula. EHP, health package. RMNCAH, Reproductive, Maternal, Newborn, Child and Adolescent Health; IMCI, Integrated Management of Childhood Illness.

Finally, figure 5 presents the change in relative share of various disease areas in the national budget distribution across districts, when allocating budgets according to EHP ‘full coverage’ and EHP ‘realistic coverage’.

This provides an insight into the key drivers behind the variation in district budgets between full EHP coverage allocations and realistic EHP coverage. Districts with relatively high HIV/AIDS burdens will benefit from higher proportional allocations, while districts with comparatively high rates of non-communicable disease will see relative funding decrease when transitioning from allocations guided by full EHP need to ‘realistic’ EHP need, resulting from higher national coverage (implementation) rates for interventions in certain disease areas. This also illustrates where the national implementation constraints lie by disease area.

## Discussion

The methods described seek to allocate public funds between districts for drugs and ORT, and therefore exclude costs relating to personnel, infrastructure or medical equipment. The methods provide estimates of district-level spending needs to support UHC without explicit consideration of the sources of funding (eg, government, donors, non-governmental organisations, patients) (Only the relative proportion of total spending to be assigned to each district is outlined, regardless of source of spending.).

The underlying assumption of our approach is that districts should be given the opportunity to deliver the standard level of health services specified in the chosen approach to UHC, given the expected level of healthcare need in their populations.^20^ There is growing consensus on the benefits of HBP definition in countries journeys towards UHC.^17^ As such, an increasing number of LMICs are explicitly defining some package of healthcare services for public provision (64 as of 2012).^32 33^ Additionally, as previously outlined, several LMICs have implemented a ‘needs’ based resource allocation formula. These formulae typically use criteria that serve as proxies for general health need such as population size, measures of supply including number of facilities or beds, socio-economic indicators such as level of social deprivation and health outcomes like mortality rates.^34 35^ Such measures are not directly related to HBPs, where they are defined. Consequently, there is a disconnect between national policy and subnational resource allocation, representing a missed opportunity. A particular example of this is Zambia, which has outlined a national predefined health benefits package (the Basic Healthcare Package). Despite the outlining of this package, however, allocation of resources to districts is based on population weighted by a deprivation index including poverty incidence, distance to facilities, ownership of capital, type of housing and disease burden. Although reference has been made for plans to explicitly link resource allocation with the benefits packages, no outline of how this would be undertaken has been put forward.^36^ Our approach, therefore, is distinct in explicitly linking the services from a predefined health benefits package to the resource allocation formula and therefore links subnational resource allocation to epidemiology and national-level healthcare planning. Further, we illustrate a pragmatic and straight-forward way to operationalise this approach. It should be highlighted that despite similarities in data requirements, these two exercises—definition of a national HBPs and development of a subnational resource allocation formulae—are distinct, in objective, process and how the data is used to inform decisions. While a growing number of LMICs have outlined a national benefits package and some have ‘needs’ based resource allocation formulae, to date, few have linked the two components.

As with all funding formula initiatives, the methods adopted were to some extent constrained by the nature and availability of relevant data, although our study was able to use a number of recently collated data sources on district-level population, disease prevalence and costs. Nevertheless, some of the data items rely on self-reporting or administrative utilisation data. These may be misleading, as they may be heavily influenced by the existing distribution of services. It would therefore be preferable to rely on estimates of disease prevalence and incidence arising from national statistical models. Although modelled estimates of disease prevalence are less direct than reported levels, they are prepared on a consistent basis and alleviate the concerns of systematic biasses that may arise from using administrative data. Future analysis may therefore consider use of modelled rather than reported prevalence for some diseases. Further, Malawi lacked information on district variations in PIN. Therefore, we use a national estimate of PIN for interventions, thereby assuming it constant across districts. One solution to this was to define as specifically as possible the target population for each intervention, where district-level variation was available. Doing so reduces the influence that the population in need has on the final calculation of patients requiring provision of an intervention.

Development of the formula benefitted from existing estimates of the unit costs of treatment that fall on the drugs budget.^27^ This obviated the need for econometric estimates of expected costs, the approach adopted in many countries without an explicit health benefits package. While this was a major benefit of the Malawi administrative arrangements, it may nevertheless be the case that there exist some legitimate variations in the unit costs of delivering services across the country. We were not able to model any such variations. However, because the formula is designed only to allocate drugs and ORT budgets, district variation in cost is likely to be less material than if personnel costs were under consideration.

Estimates of spending need were initially calculated without reference to actual national resources available, and then scaled back to conform to the available budget. Each district therefore receives an equal proportionate reduction in the allocation implied by the funding formula. That is, the approach assumes that the reductions in coverage required due to the funding shortfall are, financially, shared equally across the districts. There are other valid methods of sharing the burden of keeping within the available budget. For example, there could be a requirement that some priority treatments are funded assuming 100% coverage, involving larger proportionate reductions in other service funding, implying a ‘within EHP prioritisation’ process.

We implicitly assume that funding from other sources (eg, donors) can be flexibly incorporated into aggregate district drug and ORT budgets. However, given that the MoH has only limited influence over these funds, the constraints in what additional funds are committed to could be explicitly modelled.^20 21^ For instance, an alternative suggested approach for highly donor dependent countries is to first account for donor funding allocations in government budgetary allocation formulae. This, however, places an undue burden on recipient country governments to continuously collate data on donor allocations, a significant task in itself. Donor funding can also be highly unpredictable with resources budgeted for at the beginning of the fiscal year not equalling those subsequently disbursed. This can be seen in Malawi with a comparison of prospective Resource Mapping exercises with retrospective National Health Accounts. Finally, it operates under the working assumption that recipient governments should be responsive to donor demands rather than vice versa. It was, therefore, deemed preferable to calculate relative funding needs across districts without accounting for donor allocations. Governments can then use these relative needs to allocate the funds over which they have direct control according to the principles of equity and work towards aligning donor funding according to government priorities. However, when applying the model specifically to assess government spending need, if desired, adjustments can be made for receipts from other sources, as has been highlighted by the existing literature.^4 6^ Further, all variants assume that district populations are geographically captive, with district of residence perfectly related to district in which care is sought. In reality there is migration in accessing care between districts. Information on the net flow of patients between districts could be incorporated into the formulae to inform the transfer of funds.^23^

The budgets covered by the formula do not include personnel costs. This may have consequences for the drugs and ORT formula if, for example, shortages of personnel in a district impact coverage of some EHP treatments. Ideally, and over time, we would recommend that personnel costs are included in the formula (even if they continue to be paid by the national government) as this will help to align the separate revenue streams and potentially inform other planning policies.^22^ The third variant of ‘realistic’ coverage could serve as a basis for ensuring that funds are used most efficiently in the short-term, by directing them towards districts that can currently secure higher levels of access and coverage. However, such an approach clearly compromises equity objectives of securing equal access and is unlikely to be sustainable in the longer-term.

The results imply major changes in budgetary allocations for most districts. It is likely that immediate implementation of changes of such magnitude would be managerially and politically infeasible. Therefore, implementation of any new funding formula will probably require separate development of a ‘pace of change’ policy, which limits the year-on-year losses and gains to individual district budgets. The specification of such a policy is more a political than a technical undertaking and would best be developed through a dialogue between political and technical advisors. The new formulae can, however, indicate to which districts any new money might be best directed to promote system objectives.

In this study, we have demonstrated how it is feasible to develop a formulaic approach towards allocation that is consistent with the underlying goals of UHC—to facilitate universal access to an affordable set of services of adequate quality.^37^ Much of the discussion of UHC has, to date, taken place at national level focussing on the financial gap which requires closing through resource mobilisation. However, there remain substantial efficiency gains that could go a long way to achieving UHC through making better use of available funds.^38^ In circumstances where aggregate costs of HBP provision exceed the national budget it will never be possible to finance the full HBP, illustrating that increasing resources in many settings will be necessary in order to achieve UHC. However, a prudent first step is to ensure existing resources are used effectively. Further, with many LMICs pursuing a model of decentralised health systems, resource allocation formulae become increasingly important in the pursuit of UHC. If a country’s national and subnational plans are guided by the principals of UHC, then such a resource allocation formulaic approach can align the funding of subnational authorities with the chosen model of UHC.

While the study has explicitly examined the case of subnational health resource allocation in Malawi, it is not intended to provide a specific conclusion of a final formulae that should be applied in this case. The overarching recommendation of the study is to argue the merits of linking subnational resource allocation formulae to nationally-defined HBPs, while also illustrating how this can be done in a straight-forward manner. In that sense, the results arising from the application of the formulae should not be regarded as conclusive. Rather they represent a starting point for policymakers in Malawi to challenge, validate and refine the underlying methods, assumptions and data and ultimately reach a policy consensus on the most suitable way of calculating district budgetary allocations. The approach presented here can serve as a useful mechanism for informing that dialogue, and the principles and approaches are relevant to a wide range of countries pursuing UHC. Most countries have access to data sources that can inform the implementation of subnational health resource allocation formulae. The methods outlined therefore represent an important and feasible step for LMICs in moving towards UHC.
