# Supplementary material for: Allocating resources to support universal health coverage: development of a geographical funding formula in Malawi
Source: BMJ Glob Health. 2020 Sep 15;5(9):e002763. doi: 10.1136/bmjgh-2020-002763 (PMC7493092; doi:10.1136/bmjgh-2020-002763)
Supplement: Supplementary data [file bmjgh-2020-002763supp001.pdf]

Supplementary Files

Suppl. Appendix 1: The Malawian Essential Health Package & basis for service cost calculations

The definition of a standard health benefits package, delivered free at the point of access, was first developed in 2004 in the Programme of Work (2004-2010). The package was revised in 2010 and delivered under the Health Sector Strategic Plan (2010-2016) with the current EHP being defined in 2017 for delivery under the Health Sector Strategic Plan II (2017-2022).

The objective of the EHP is stated as "to ensure timely universal free access to a quality Essential Health Package, irrespective of ability-to-pay, to all the people in Malawi." (HSSP II, 2017-2022). The HSSPII explicitly acknowledges that the cost of fully providing the EHP exceeds the resources available for its provision. A number of related issues are outlined which could impact EHP provision including; lack of awareness of the EHP among stakeholders, lack of EHP policy enforcement, inequalities in utilisation and not linking health system inputs to the EHP.

Table S1 below outlines the interventions included in the EHP and the level at which they are provided in addition to the calculation for the service costs for each individual intervention. The calculations were made according to the target populations for each district.

Table S1: Malawi's EHP as defined in the HSSP II

Category	Intervention Package	Intervention	Level of Care	Target population	Population in Need (PIN)	Coverage	Unit cost (US$ 2016)
RMNCH	ANC Package	Tetanus toxoid (pregnant women)	Community/Primary/Secondary	Pregnant women	1.00	0.93	0.06
		Deworming (pregnant women)	Community/Primary/Secondary	Pregnant women	0.29	1.00	0.05
		Daily iron and folic acid supplementation (pregnant women)	Community/Primary/Secondary	Pregnant women	0.31	0.79	0.67
		Syphilis detection and treatment (pregnant women)	Community/Primary/Secondary	Pregnant women	1.00	1.00	0.46
		IPT (pregnant women)	Community/Primary/Secondary	Pregnant women	1.00	0.47	0.18
		ITN distribution to pregnant women	Community/Primary/Secondary	Pregnant women	1.00	0.63	0.31
		Urinalysis (4 per pregnant woman)	Primary/Secondary	Pregnant women	1.00	0.55	6.26
		Injectable	Community/Primary/Secondary				0.50

Category	Intervention Package	Intervention	Level of Care	Target population	Population in Need (PIN)	Coverage	Unit cost (US$ 2016)
	Modern Family Planning	IUD	Primary/Secondary				0.16
		Implant	Primary/Secondary				1.10
		Pill	Community/Primary/Secondary				1.05
		Female sterilization	Secondary				19.53
		Male condom	Community/Primary/Secondary	Male adults 15-49 years	1.00	0.53	3.04
	Delivery Package	Clean practices and immediate essential new-born care (in facility)	Primary/Secondary	Births	1.00	-	1.35
		Active management of the 3rd stage of labour	Primary/Secondary	Pregnant women	1.00	0.67	0.06
		Management of eclampsia (Magnesium sulphate, Methyldopa, Nifedipine, Hydralazine)	Primary/Secondary	Pregnant women	0.018	0.873	4.77
		Management of pre-eclampsia	Primary/Secondary	Pregnant women	0.0218	0.8733	4.77
		Neonatal resuscitation (institutional)	Primary/Secondary	Births	0.01	1.00	8.13
		Caesarean section with indication	Secondary	Pregnant women	0.005	0.897	21.05
		Caesarean section with indication (with complication)	Secondary	Pregnant women	0.0009	0.8972	49.44
		Vaginal delivery, skilled attendance	Primary/Secondary	Pregnant women	0.8449	0.8972	4.28
		Vaginal delivery, skilled attendance (with complications)	Primary/Secondary	Pregnant women			3.69
		Management of obstructed labour	Primary/Secondary	Pregnant women	0.10	1.00	30.21
		Newborn sepsis - full supportive care	Primary/Secondary	Births	0.12	0.87	9.70
		Newborn sepsis – injectable antibiotics	Primary/Secondary	Births	0.09	0.87	0.73
		Antenatal corticosteroids for preterm labour	Primary/Secondary	Pregnant women	0.18	0.50	6.39
		Maternal sepsis case management	Primary/Secondary	Pregnant women	0.07	0.82	51.25
		Cord Care Using Chlorhexidine	Primary/Secondary	Births	1.00	0.76	0.07

Category	Intervention Package	Intervention	Level of Care	Target population	Population in Need (PIN)	Coverage	Unit cost (US$ 2016)
		Hysterectomy	Primary/Secondary				
		Post-abortion case management	Secondary				12.34
		Treatment of antepartum haemorrhage	Primary/Secondary	Pregnant women			
		Treatment of postpartum haemorrhage	Secondary	Pregnant women	0.039	0.640	16.96
		Antibiotics for pPRoM	Primary/Secondary	Pregnant women	0.07	0.81	7.74
Vaccine Preventable diseases	Essential Vaccines Package	Rotavirus vaccine	Community/Primary/Secondary	Children < 1	1.00	0.94	2.19
		Measles Rubella vaccine	Community/Primary/Secondary	Children < 1	1.00	0.95	0.65
		Pneumococcal vaccine	Community/Primary/Secondary	Children < 1	1.00	0.92	2.97
		BCG vaccine	Community/Primary/Secondary	Children < 1	1.00	0.98	0.19
		Polio vaccine	Community/Primary/Secondary	Children < 1	1.00	0.87	0.11
		DPT-Heb-Hib / Pentavalent vaccine	Community/Primary/Secondary	Children < 1	1.00	0.95	1.83
		HPV vaccine	Community/Primary/Secondary	Females 9-13 years	2	0.67	0.71
Malaria	First Line uncomplicated Malaria treatment	Uncomplicated (adult, <36 kg)	Community/Primary/Secondary	Population >15 who suffered fever or malaria in past 2 weeks	1.0	0.33	1.75
		Uncomplicated (adult, >36 kg)	Community/Primary/Secondary	Population >15 who suffered fever or malaria in past 2 weeks	1.00	0.67	2.00
		Uncomplicated (children, <15 kg)	Community/Primary/Secondary	Population 0-4 who suffered fever or malaria in past 2 weeks	1.00	0.59	22.41
		Uncomplicated (children, >15 kg)	Community/Primary/Secondary	Population 0-4 who suffered fever or malaria in past 2 weeks	1.00	0.41	23.24

Category	Intervention Package	Intervention	Level of Care	Target population	Population in Need (PIN)	Coverage	Unit cost (US$ 2016)
	Complicated Malaria treatment	Complicated (adults, injectable artesunate)	Primary/Secondary	Population >15 who suffered fever or malaria in past 2 weeks	1.00	1.00	2.79
		Complicated (children, injectable artesunate)	Primary/Secondary	Population 0-4 who suffered fever or malaria in past 2 weeks	1.00	1.00	1.33
	Malaria Diagnosis	RDTs	Community/Primary/Secondary				
		Microscopy for Malaria	Primary/Secondary				
Integrated management of childhood illnesses (IMCI)	ARIs	Pneumonia treatment (children)	Community/Primary/Secondary	Children 1-59 months	0.03	0.77	0.41
		Treatment of severe pneumonia (Oxygen)	Primary/Secondary	Children 1-59 months	0.0007	0.7713	5.54
	Diarrhoeal Disease	ORS	Community/Primary/Secondary	Children 0-59 months	2.22	0.70	0.05
		Zinc	Community/Primary/Secondary	Children 1-59 months	2.22	0.46	0.18
		Treatment of severe diarrhoea (IV Fluids)	Primary/Secondary	Children 1-59 months	0.02	1.00	0.45
	Nutrition	Community management of nutrition in under-5 - Plumpy Peanut	Community/Primary				
		Community management of nutrition in under-5 - micronutrient powder	Community/Primary				
		Community management of nutrition in under-5 - vitamin A	Community/Primary				
	Malaria Diagnosis	RDTs for under-5	Community/Primary				
Community Health	Community Health Package	Growth Monitoring	Community/Primary				0
		Vermin and Vector Control & Promotion	Community/Primary				0
		Disease Surveillance	Community/Primary				0
		Community Health Promotion & Engagement	Community/Primary				0
		Village Inspections	Community/Primary				0

Category	Intervention Package	Intervention	Level of Care	Target population	Population in Need (PIN)	Coverage	Unit cost (US$ 2016)
		Promotion of hygiene (hand washing with soap)	Community/Primary				0
		Promotion of Sanitation (latrine refuse, drop hole covers, solid waste disposal, hygienic disposal of children's stools)	Community/Primary				0
		Occupational Health Promotion	Community/Primary				0
		Household water quality testing and treatment	Community/Primary				
		Home-based care of chronically ill patients	Community/Primary				0
		Child Protection	Community/Primary				0
NTDs	Treatment and MDA	Schistosomiasis mass drug administration	Community/Primary				0.01
		Case finding and treatment of Trypanosomiasis	Primary				
		Trachoma mass drug administration	Community/Primary				0.53
HIV/AIDS	HIV Prevention	Cotrimoxazole for children	Community/Primary/Secondary				5.15
		PMTCT	Community/Primary/Secondary	Pregnant women with HIV/AIDS	1.00	0.85	11.35
	HIV Testing	HIV Testing Services (HTS)	Community/Primary/Secondary				4.73
	HIV Treatment	HIV Treatment for all ages – ART & Viral Load	Community/Primary/Secondary	HIV prevalence	1.00	1.00	7.64+95.82
Nutrition		Vitamin A supplementation in pregnant women	Community/Primary/Secondary	Pregnant women	0.14	0.67	2.31
		Management of severe malnutrition (children)	Community/Primary/Secondary	Number of children <5 -3 SD below weight-for-age	1.00	0.7333	89.16
		Deworming (children)	Community/Primary/Secondary	Children 0-14 years	1.00	0.72	0.17
		Vitamin A supplementation in infants and children 6-59 months		Number of children <5 months not fed minimum dietary			

Category	Intervention Package	Intervention	Level of Care	Target population	Population in Need (PIN)	Coverage	Unit cost (US$ 2016)
			Community/Primary/Secondary	diversity (assuming same proportions as children 6-23 months	1.00	0.89	0.09
TB		Isonized Preventive Therapy for children in contact with TB patients	Primary/Secondary				3.29
		First line treatment for new TB Cases for adults	Primary/Secondary	Total population >15	0.00227	0.79	20.02
		First line treatment for retreatment TB Cases for adults	Primary/Secondary	Total population >15	0.00227	0.12	86.15
		First line treatment for new TB Cases for children	Community/Primary/Secondary	Total population <15	0.00227	0.09	33.88
		First line treatment for retreatment TB Cases for children	Community/Primary/Secondary	Total population <15	0.00227	0.005	75.32
		Case management of MDR cases	Primary/Secondary				858.82
	TB Testing	LED test	Primary/Secondary				0.24
		Xpert test	Primary/Secondary				9.83
		MGIT test	Primary/Secondary				31.75
		LJ test	Primary/Secondary				0.21
NCDs		Treatment of Injuries (Fracture & dislocation)	Primary/Secondary	Total population	0.0008	1.00	51.63
		Treatment of Injuries (Blunt trauma / soft tissue injuries)	Primary/Secondary	Total population	0.0004	1.00	9.74
	Mental Health treatment	Basic psychosocial support, advice, and follow-up	Community/Primary/Secondary				
		Anti-epileptic medication	Community/Primary/Secondary	Population with epilepsy	1.00	0.05	6.96
		Treatment of depression (first line)	Community/Primary/Secondary	Of those chronically ill proportion with mental illness	1.00	0.02	27.15
		Testing of pre-cancerous cells (vinegar)	Primary/Secondary				

Category	Intervention Package	Intervention	Level of Care	Target population	Population in Need (PIN)	Coverage	Unit cost (US$ 2016)
	Diabetes treatment	Diabetes Type I	Primary/Secondary	Adults 40+	0.009	0.283	247.78
		Diabetes Type II	Primary/Secondary	Adults 40+	0.056	0.283	102.56
		Hypertension	Primary/Secondary	Adults 40+	0.329	0.25	8.56
Oral Health	Tooth pain treatment	Management of severe tooth pain, tooth extraction	Primary/Secondary	People 4+	0.04	1.00	0.30
		Management of mild tooth pain, tooth filling	Primary/Secondary	People 4+	0.02	1.00	2.49

Data on patient populations comes from a mid-term re-costing of the Health Sector Strategic Plan (2011-2016) undertaken by Clinton Health Access Initiative (CHAI) in 2014. For each intervention the target population (e.g. pregnant women, women in need of PMTCT, etc.) was identified. The cost of each intervention was calculated using a bottom-up ingredients based approach but only reflects drug and supply inputs rather than a cost per patient treated.

Suppl. Appendix Table S2: Selected target population calculations

Target population	Data source & calculation	Assumptions
Pregnant women	Data:<br>- Females 15-49 years (Population & Housing Census, 2008)<br>- % women aged 15-49 pregnant (Demographic & Household Survey, 2015/16)<br><br>Calculation:<br><br>$\text{Females } 15-49 \text{ years} \times \left(\frac{\%\text{ women aged } 15-49 \text{ currently pregnant}}{100}\right)$	- No women outside the age range 15-49 are pregnant or the proportion of women pregnant outside this age range is proportional across districts.<br>- Data was not available on the percentage of women pregnant in Blantyre, Lilongwe, Mzuzu and Zomba city. The percentage from their corresponding district was used.
Births	Data:<br>- Live births (2016) (District Health Information System 2)<br><br>Calculation:<br><br>N/A	
Population >15 years who suffered fever or malaria in past 2 weeks	Data:<br>- Proportion who suffered incidence of sickness in past 2 weeks (Integrated Household Survey 4, 2016/17)<br>- Of those who suffered sickness proportion who suffered malaria or fever (Integrated Household Survey 4, 2016/17)<br>- Population >15 (Population & Housing Census, 2008)<br><br>Calculation:<br><br>$\left(\frac{\%\text{ suffering sickness}}{100}\right) \times \left(\frac{\text{Of those }\%\text{ suffering malaria or fever}}{100}\right) \times \text{population} > 15 \text{ years}$	- Assumption that the 2 week period in which the survey was taken does not suffer any systematic differences with the rest of the year which effect the geographical distribution of malaria.
Population 0-4 years who suffered fever or malaria in past 2 weeks	Data:<br>- Proportion who suffered incidence of sickness in past 2 weeks (Integrated Household Survey 4, 2016/17)<br>- Of those who suffered sickness proportion who suffered malaria or fever (Integrated Household Survey 4, 2016/17)<br>- Population 0-4 years (Population & Housing Census, 2008)<br><br>Calculation:	- Assumption that the 2 week period in which the survey was taken does not suffer any systematic differences with the rest of the year which effect the geographical distribution of malaria.

	$\left(\frac{\text{\% suffering sickness}}{100}\right) \times \left(\frac{\text{Of those \% suffering malaria or fever}}{100}\right) \times \text{population } 0-4 \text{ years}$	
Number of children <5 years -3 standard deviations below weight-for-age	Data:<br>- Children < 5 years (Population & Housing Census, 2008)<br>- Proportion of children <5 years -3 standard deviations below weight-for-age (Demographic & Household Survey, 2015/16)<br><br>Calculation:<br><br>$\text{Children} < 5 \text{ years} \times \left(\frac{\text{\% children} < 5 - 3 \text{ SD below weight} - \text{for} - \text{age}}{100}\right)$	- This target population is applied to the nutrition intervention 'management of severe malnutrition'. In the EHP tool this intervention had a target population children 1-59 months with a population in need of 1.15%. As the average proportion of children <5 -3 SD below weight-for-age across all districts is 2.5% this was deemed a usable replacement for the target population with a new assumed 100% population in need. The intention is this would capture more of the district variation in expected expenditure for this intervention.
Number of children <5 years not fed minimum dietary diversity	Data:<br>- Children < 5 years (Population & Housing Census, 2008)<br>- Proportion of children aged 6-23 months fed minimum dietary diversity (Demographic & Household Survey, 2015/16)<br><br>Calculation:<br><br>$\text{Children} < 5 \text{ years} \times \left(\frac{\text{\% children aged } 6-23 \text{ months fed minimum dietary diversity}}{100}\right)$	- Assumes the proportion of children fed minimum dietary diversity is constant for those aged 6-23 months and those aged <5 years, or at least the relative proportions across districts are constant.
HIV+ population	Data:<br>- Population 15-49 years (Population & Housing Census, 2008)<br>- HIV prevalence among population 15-49 years (Demographic & Household Survey, 2015/16)<br><br>Calculation:<br><br>$\text{Population } 15-49 \text{ years} \times \left(\frac{\text{HIV prevelance (\%) among } 15-49 \text{ years}}{100}\right)$	

Pregnant women with HIV/AIDS	Data:<br>- Females 15-49 years (Population & Housing Census, 2008)<br>- % women aged 15-49 pregnant (Demographic & Household Survey, 2015/16)<br>- HIV prevalence among population 15-49 years (Demographic & Household Survey, 2015/16)<br><br>Calculation:<br><br>$\left(\text{Females } 15-49 \text{ years} \times \left(\frac{\%\text{ women aged } 15-49 \text{ currently pregnant}}{100}\right)\right) \times \times \text{ HIV prevelance (\%) among } 15-49 \text{ years}$	
Population with mental illness	Data:<br>- Proportion of population chronically illness (Integrated Household Survey, 2016/17)<br>- Of those chronically ill, proportion with mental illness (Integrated Household Survey, 2016/17)<br>- Population (Population & Housing Census, 2008)<br><br>Calculation:<br><br>$\left(\frac{\%\text{ suffering chronic illness}}{100}\right) \times \left(\frac{\text{Of those, \% suffering mental illness}}{100}\right) \times \text{population}$	- As this target population is applied to treatment of depression there is an assumption that depression is distributed across districts in proportion to all mental illness.
Population with epilepsy	Data:<br>- Proportion of population chronically illness (Integrated Household Survey, 2016/17)<br>- Of those chronically ill, proportion with epilepsy (Integrated Household Survey, 2016/17)<br>- Population (Population & Housing Census, 2008)<br><br>Calculation:<br><br>$\left(\frac{\%\text{ suffering chronic illness}}{100}\right) \times \left(\frac{\text{Of those, \% suffering epilpsy}}{100}\right) \times \text{population}$	
